# Supplementary material for: Is the Addition of Chemotherapy to Adjuvant Radiation in Merkel Cell Cancer Beneficial? Real-World Data with Long-Term Follow-Up
Source: Cancers (Basel). 2025 Mar 11;17(6):945. doi: 10.3390/cancers17060945 (PMC11939928; doi:10.3390/cancers17060945)

Figure S1: 5-Year Disease Free Survival in Radiation and Chemoradiation Treated MCC Patients

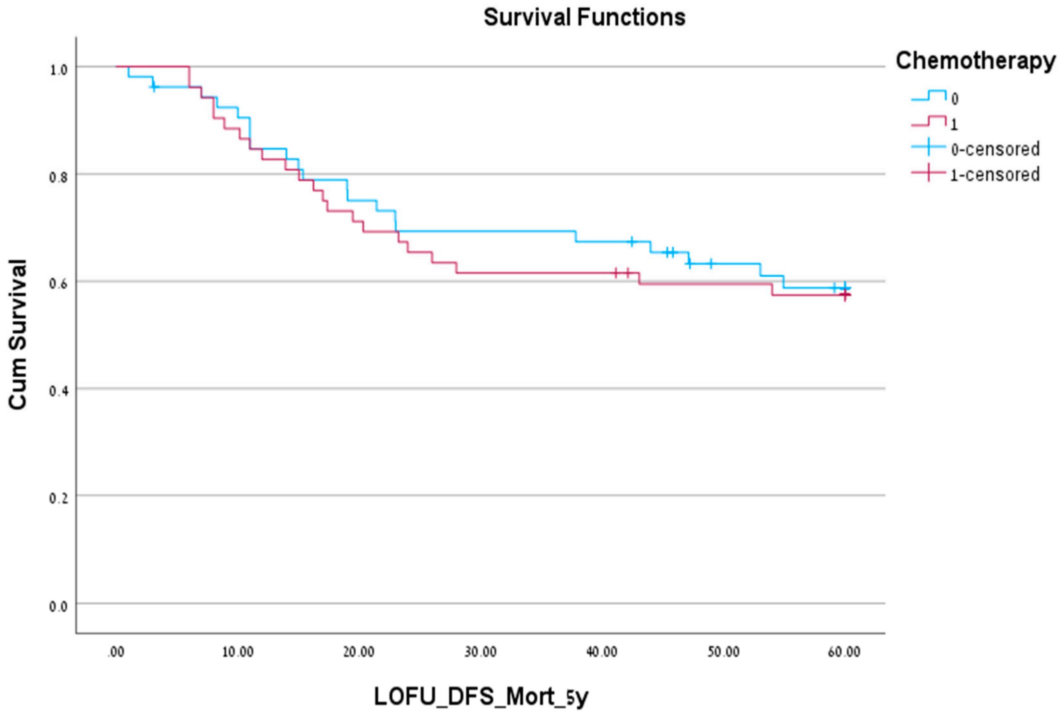

Figure S2: 20-Year Disease Free Survival in Radiation and Chemoradiation Treated MCC Patients

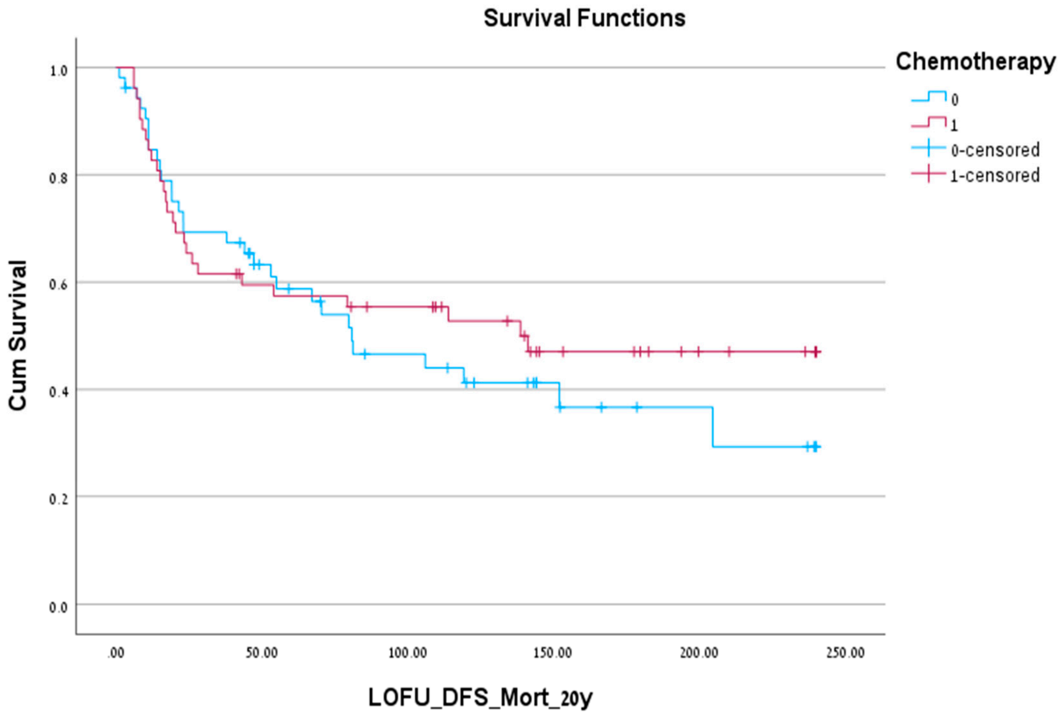

<u>Figure S3</u>: 5 and 20-year Overall Survival in Radiation and Chemoradiation Treated MCC Patients excluding unknown primary

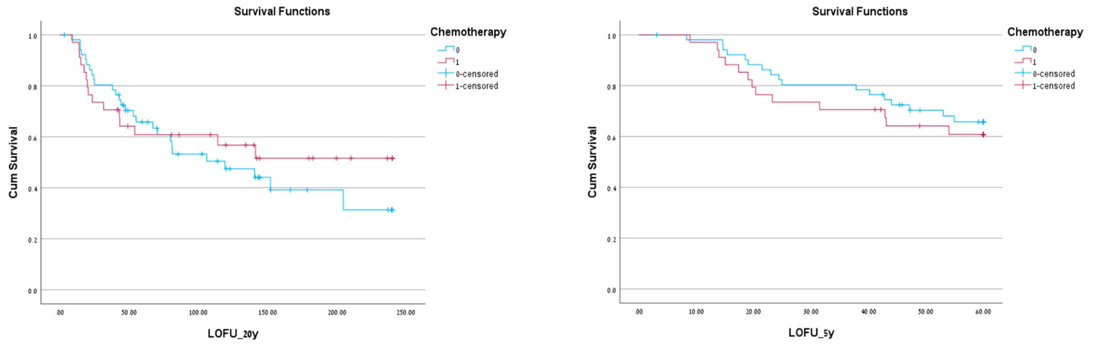

<u>Figure S4</u>: 5 and 20-year Disease-free Survival in Radiation and Chemoradiation Treated MCC Patients excluding unknown primary

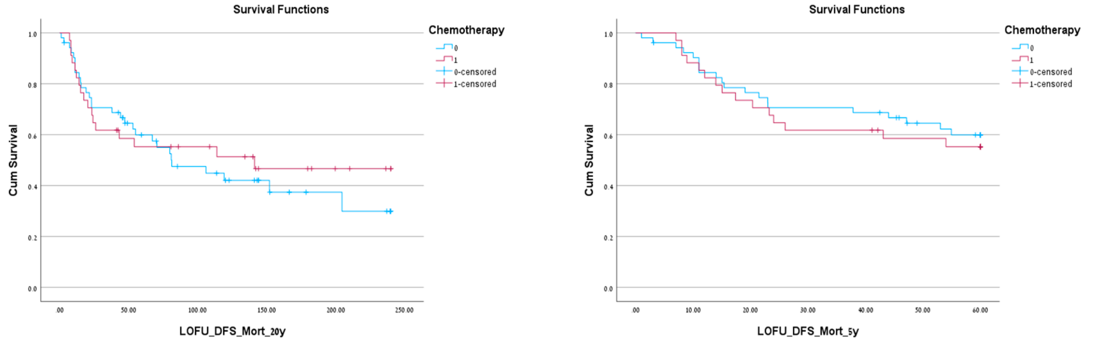

Supplement: Supplementary file 1 [file cancers-17-00945-s001.zip › cancers-3478154-supplementary.pdf]
